# Strain-tuned enhancement of ferromagnetic *T*_*C*_ to 176 K in Sm-doped BiMnO_3_ thin films and determination of magnetic phase diagram

**DOI:** 10.1038/srep43799

**Published:** 2017-03-03

**Authors:** Eun-Mi Choi, Josée E. Kleibeuker, Judith L. MacManus-Driscoll

**Affiliations:** 1 Department of Materials Science, University of Cambridge, 27 Charles Babbage Road, Cambridge, CB3 0FS, UK

## Abstract

BiMnO_3_ is a promising multiferroic material but it’s ferromagnetic *T*_*C*_ is well below room temperature and the magnetic phase diagram is unknown. In this work, the relationship between magnetic transition temperature (*T*_*C*_) and the substrate induced (pseudo-) tetragonal distortion (ratio of *out-of-plane* to *in-plane* lattice parameters, *c/a*) in BiMnO_3_ thin films, lightly doped to optimize lattice dimensions, was determined. For *c/a* > 0.99, hidden antiferromagnetism was revealed and the magnetisation versus temperature curves showed a tail behaviour, whereas for *c/a* < 0.99 clear ferromagnetism was observed. A peak *T*_*C*_ of up to 176 K, more than 70 K higher than for bulk BiMnO_3_, was achieved through precise strain tuning. The *T*_*C*_ was maximised for strong tensile *in-plane* strain which produced weak octahedral rotations in the *out-of-plane* direction, an orthorhombic-like structure, and strong ferromagnetic coupling.

Strong coupling between magnetism and ferroelectricity in perovskite oxides (*ABO*_3_) has drawn increasing interest because of the potential for magnetoelectric devices for highly sensitive magnetic sensors and low energy random access memory (RAM). BiMnO_3_ (BMO) has been extensively investigated because it is one of the rare multiferroics that possesses both ferromagnetism (FM) and ferroelectricity (FE). However, its maximum reported magnetic transition temperature, *T*_*C*_, is only ~100 K^1^,^2^. In addition, recent theoretical and experimental studies have shown that bulk BMO has a centrosymmetric monoclinic structure (*C2/c*) which means the ground state of BMO is not ferroelectric (FE)^3^. Solovyev *et al*. proposed that hidden antiferromagnetic (AFM) ordering is responsible for the ferroelectricity^4^. These studies have shown the potential of BMO to be a multiferroic, but the combination of strong FM and FE are difficult to achieve.

The magnetic behaviour of BMO is complex owing to competition between FM and AFM coupling. The 6 *s*^*2*^ lone pair character of Bi^3+^ results in a much higher Jahn-Teller (*JT*) structural distortion than, for example, in A-type AFM LaMnO_3_ (LMO)^5^,^6^,^7^,^8^,^9^,^10^. According to the Goodenough-Kanamori rules, this distortion breaks the degeneracy of the *e*_*g*_ orbitals as shown in Fig. 1a. The half-filled 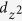 (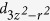) orbitals point towards the empty 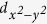 (
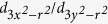
) orbitals on the next manganese and such interactions are ferromagnetic. In addition, the superexchange interaction between Mn^3+^ ions becomes antiferromagnetic when both *e*_*g*_ orbitals point perpendicular to the bond direction despite the interaction between 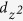 and 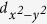 orbitals. On the other hand, the ferromagnetic interaction occurs if one of the *e*_*g*_ orbitals points along the bond direction^9^. In BMO, the interaction between the 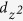 and 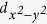 orbitals happens along both *in-plane* and *out-of-plane* directions and leads to three-dimensional (3D) ferromagnetic interactions (see Fig. 1b). However, there are also AFM interactions along the ab-plane (*in-plane*) direction^7^,^8^,^9^,^10^. The special *e*_*g*_ orbital ordering in BMO is caused by the 6*s*^*2*^ lone pair character of Bi^3+^ that leads to *A* cations displacements along the <111> direction^10^. The 3D magnetic interactions in BMO are very different to LMO where ferromagnetic planes alternate antiferromagnetically along *out-of-plane* (A-type AFM).

Strain in the structure (whether induced isostatic pressure applied to bulk samples or biaxial pressure applied in thin films by heteroepitaxial growth) can cause octahedral rotations that change the Mn-O-Mn bond lengths and angles. The 3D magnetic state of BMO is very sensitive to changes in Mn-O-Mn bond lengths and angles. Even as low as a ~1° bond angle change radically alters the magnetic properties^7^. Overall, long-range FM coupling dominates over AFM coupling, but ‘hidden’ AFM remains and is revealed under certain strain conditions^7^,^8^,^11^. This explains why *T*_*C*_s of between 50 and 100 K have been observed when films are grown on different substrates^12^,^13^,^14^. Therefore, precise tuning of the Mn-O-Mn bond lengths and angles is critical for engineering the FM properties. It should be noted, however, that when the FM properties are optimised, the FE properties are not necessarily optimised^1^,^11^,^13^,^15^,^16^,^17^.

Recently, the improved multiferroic properties of BMO thin films by independently tuning the *in-plane* and *out-of-plane* bond lengths, using a combination of isostatic chemical pressure and biaxial mechanical pressure were demonstrated. The chemical pressure was induced by low-level Sm doping of the *A*-site to decrease the overall cell volume and the biaxial mechanical pressure was induced using epitaxial strain. The contraction of the BiMnO_3_ lattice with Sm, together with the *in-plane* lattice extension by growth on SrTiO_3_ (*a* = 3.905 Å) enabled optimization of both the *in-plane* and *out-of-plane* lattice dimensions. An enhancement of FM interactions in the *out-of-plane* direction by reducing the *out-of-plan*e lattice parameter and a reduction of AFM interactions *in-plane* by increasing the *in-plane* lattice parameter were shown. An increase in the *T*_*C*_ to 140 K was achieved, as well as FE behaviour at room temperature (RT). However, since the aim of the earlier work was to measure FE properties, the films were grown relatively thick (~200 nm), in order for the FE leakage to be minimised, and so uniform straining of the Bi_1−x_Sm_x_MnO_3_ (x = 0.15, BSMO) film was lost. Consequently, a range of strain states in the films meant that large AFM component remained in the films and a strong tail behaviour in the magnetic transition was observed together with an AFM transition at ~30 K, indicating the competition between the AFM and FM interactions^9^,^11^,^13^,^18^,^19^. This result is in agreement with the suggestion that hidden AFM ordering is responsible in FE materials^4^.

The main aim of this work is to achieve a range of different strain states in BSMO thin films so as to determine the conditions for optimising its magnetic properties, i.e. to minimise the AFM component and maximise the FM component. A secondary aim is to shed light on the magnetic phase diagram. The ferroelectric properties of the very thin films of this work were not measured. This is because thin film oxide perovskite ferroelectrics (no matter their composition) show leaky behaviour, and the 20 nm BSMO films studied here are no exception. Also, the bottom electrode, SrRuO_3_, which would need to be used on the different insulating substrates, may induce unexpected octahedral rotations in the BSMO films^20^. On the other hand, we have already demonstrated that relatively thick BSMO films grown on SrTiO_3_ show excellent ferroelectric properties at RT^18^. Further work is required to measure the FE properties without changing the octahedral rotations from SrRuO_3_ (or any other bottom electrode film), for example by using second-harmonic generation studies of films grown directly on insulating substrates.

Very thin films (~20 nm) of BSMO were grown on a range of different substrates. It was necesssary to grown very thin films (~20 nm) to provide a near-uniform strain state. The structural distortion of the films was measured using conventional x-ray diffraction (XRD). This gives an indirect assessment of the octahedral rotations: the further the (pseudo-) tetragonal distortion (*c/a*) is from 1, the greater level of rotation^20^,^21^,^22^,^23^. While synchrotron XRD would be optimum to determine bond angles^21^,^22^, these measurements were not performed here since the aim of work to demonstrate property enhancement though careful materials engineering.

The key finding of our work is that by engineering an optimal structural distortion into BSMO films, enhanced FM *T*_*C*_’s with clear transitions, up to 176 K, can be achieved. At the same time, by engineering a range of structural distortions into films, the magnetic phase diagram was determined.

Two different experiments were undertaken. The first one explored the magnetic properties of the films of constant thickness (20–24 nm) on substrates with different lattice mismatch. Thicknesses of ~20 nm were low enough to give coherent growth with little relaxation of strain but thick enough to give a sizeable magnetic signal. The second study focused on the magnetic properties of films of a range of thicknesses (10, 20 and 200 nm) grown on SrTiO_3_ (STO). Films were grown on orthorhombic, paramagnetic (110) DyScO_3_ (DSO), (110) GdScO_3_ (GSO), (110) NdGaO_3_ (NGO) substrates, on cubic, non-magnetic (001) TiO_2_-terminated STO, and on rhombohedral, non-magnetic (001) LaAlO_3_ (LAO) substrates. All substrates were thermally treated to show well defined terraces before deposition. The lattice mismatch values between the BSMO and the various substrates are shown in Fig. 2a. BSMO lattice parameter is assumed from a pseudo-cubic cell volume of 58.8 Å^3^ 18,24,25.

## Results and Discussion

Figure 2b shows *2θ* − *ω* XRD patterns of 20–24 nm BSMO films grown on the various substrates. The films were fully epitaxial and no impurity phases were observed (Fig. 2b). Clear thickness fringes for all these films were observed (except for LAO), indicating the high crystal quality. From NGO to GSO, the (001)_pc_ peak of the BSMO shifts to a higher 2*θ* angle indicating a reduction of the *out-of-plane* lattice parameter, which is consistent with the *in-plane* strain becoming more tensile across the series, as expected. The inset of Fig. 2b shows an AFM height image of a typical film grown on STO. A homogeneous surface morphology of aligned rectangular faceted grains was observed.

Figure 2c shows reciprocal space maps (RSMs) around the (332) peak of DSO, GSO and NGO, and (103) of STO and LAO. The coincidence of the substrate and film *in-plane* reciprocal lattice vector values (Q_in_) from NGO to GSO indicates that the films are fully *in-plane* strained to the substrates. The shift of the film peaks to higher Q_in_ values indicates an increase of the *in-plane* lattice parameter which is expected as the substrate lattice parameter increases (Fig. 2c). On the other hand, consistent with Fig. 2b, the *out-of-plane* reciprocal lattice vector (Q_out_) shifts to higher values as the substrate lattice parameters increase. The film on LAO is fully relaxed, showing that the growth is incoherent for such large *in-plane* compressive substrate strain (−2.32%). This also explains the absence of thickness fringes in the *out-of-plane* spectra for BSMO on LAO (Fig. 2b).

A summary of lattice parameters, cell volumes, (pseudo-)tetragonal distortions (the ratio between *out-of-plane* and *in-plane* lattice parameters, *c/a*), crystal structures, FM transition temperatures (*T*_*C*_) and remanent magnetic moment (*M*_*r*_) of the ~20 nm BSMO films are shown in Table 1.

For all the films, the BSMO cell volume is significantly reduced compared to the bulk BMO value of 61.5 Å^3^. This is consistent with the partial replacement of Bi^3+^ by the smaller Sm^3+^ ion^18^. Except for BSMO on GSO (60.4 ± 0.2 Å^3^), the pseudo-cubic cell volume of all the BSMO films was around 58.8 ± 0.3 Å^3^. The increase in unit-cell volume on GSO is likely due to the large biaxial tensile *in-plane* strain (+2.4%), which cannot be compensated for by elastically reducing the *out-of-plane* lattice parameter to such a large extent, and so instead other *out-of-plane* strain relaxation mechanisms must come into play, e.g. compositional modification such as oxygen loss or cation non-stoichiometry. This is in contrast with BSMO on LAO. Here, there is a similar strain level but it is of opposite sign (−2.32%). Unlike BSMO on LAO, GSO *can* maintain *in-plane* straining because it is possible to strengthen the Mn-O-Mn bonds. On the other hand, for the films on LAO, it is not possible to decrease the 〈Mn-O-Mn〉 angle sufficiently to accommodate the *in-plane* strain.

For NGO, STO and DSO substrates which produce more moderate *in-plane* strain, *cf*. LAO or GSO, the *out-of-plane* lattice parameters are increasingly reduced across the series, as the *in-plane* lattice parameter is increased. This is as expected from simple elastic deformation arguments. The crystal structures of the BSMO films were determined from the asymmetric RSMs at various phi-angles (See Fig. 2c, and Supporting Information). The films on STO and LAO were found to be tetragonal, while the films on DSO, GSO and NGO had an orthorhombic-like structure.

To investigate the effect of biaxial strain on the magnetic properties of the BSMO films, field cooled *M* − *T* measurements were undertaken. Herein, normalised *M* − *T* curves are shown because these are measured under two different conditions: BSMO on STO and LAO were measured at a field of 200 Oe whereas *M* − *T* curves of BSMO on DSO, GSO and NGO were measured at 0 Oe after field cooling at 1 T. The two conditions were necessary because of very high paramagnetic background of DSO, GSO and NGO substrates. For the same reason, magnetic hysteresis curves are not shown. Instead, in Table 1, *M*_*r*_ at 10 K is shown for the different films.

Figure 3 shows the *M* − *T* curves of the BSMO films on the various substrates. The data can be separated into two different types of curve. The first type shows a tail behaviour. This is found for BSMO on LAO and NGO. Such a behaviour has been reported before for BMO films and indicates the competition between AFM and FM interactions^9^,^11^,^13^,^19^. When the AFM interactions become dominant, the tail behaviour becomes stronger^19^. In addition to the tail behaviour, the BSMO films on LAO and NGO substrates also show clear AFM transitions at ~30 K, which means the hidden AFM is revealed at low temperature^18^.

The second type of curve shows a clear FM transition and no tail behaviour. This was the case for BSMO on STO, DSO and GSO. Here, *T*_*C*_s ranged from 80 K on GSO to 160 K on DSO, the latter value being enhanced by 60 K compared to strained BMO films^13^,^14^. For BSMO on DSO and GSO, the magnetic moments increase at low temperature in spite of the clear FM transitions being observed. This is not consistent with a standard FM feature where the magnetic moment saturates at low temperature. The results indicate the presence of AFM coupling^26^,^27^,^28^. For BSMO on STO, a ferromagnetic transition at 150 K is observed with a clear AFM transition at ~30 K also observed. The explanation of these two different magnetic behaviours is discussed later.

To understand the relation between magnetic behaviour and strain, *T*_*C*_ and *T*_*N*_ are plotted versus the (pseudo-)tetragonal distortion (*c/a*), as shown in Fig. 4. A clear and very sensitive relationship between the magnetic behaviour of BSMO and the (pseudo-) tetragonality of the unit cell is observed. First of all, for *c/a* < 0.99, a clear FM transition without tail behaviour is obtained. For *c/a* > 0.99, hidden AFM is obtained and is manifest as a tail behaviour in the *M* − *T* curves. The strong relationship between magnetic structure and *c/a* is consistent with other manganites^26^,^29^,^30^,^31^,^32^,^33^. For example, for La_0.5_Sr_0.5_MnO_3_, by varying the *c/a* ratio from 0.97 to 1.06, the magnetic phase changes from A-type AFM to FM to C-type AFM^29^. According to recent theoretical calculations on LaMnO_3_, an insulating FM phase is formed under small tensile strain whereas a metallic FM phase and an A-type AFM phase is formed under large compressive and tensile strain, respectively^34^. The sensitivity of the magnetic behaviour with *c/a* ratio in manganites is caused by the change of the *JT* distortion of the MnO_6_ octahedra and the change of the Mn-O-Mn bond lengths and angles.

To understand why *T*_*C*_ increases when the *c/a* ratio is reduced, it is necessary to understand more about the precise crystal structure and the MnO_6_ octahedral rotations. Minor strained and relaxed BSMO films were found to have a tetragonal crystal structure, indicating either *a*^0^*a*^0^*c*^−^ or *a*^0^*a*^0^*c*^+^ octahedral rotation patterns (Glazer’s notation)^35^. On the other hand, when the films are grown under large strain, either tensile or compressive, the film crystal structure is orthorhombic-like, as determined by doing x-ray scans around the (103)_pc_ peaks along the four pseudo-cubic *in-plane* directions (see Supporting Information). This indicates that the oxygen octahedra have an *a*^−^*a*^−^*c*^+^ rotation pattern.

From previous studies of strained oxide perovskites, A*B*O_3_, the degree of rotation along both the *in-plane* and *out-of-plane* axes depends strongly on the amount and sign of the strain^20^,^21^. Hence, under compressive *in-plane* strain (*c/a* > 0.98), there are strong rotations along the *out-of-plane* axis, in combination with weaker rotations along the *in-plane* axis^36^. Under tensile strain (*c/a* < 0.98), it is the other way around: strong rotations occur along the *in-plane* axes and weaker rotations along the *out-of-plane* axis. The change in rotation patterns and the degree of the rotations impact the 〈Mn-O-Mn〉 bond angles and, as a result, the magnetic behaviour.

**Figure 1:**
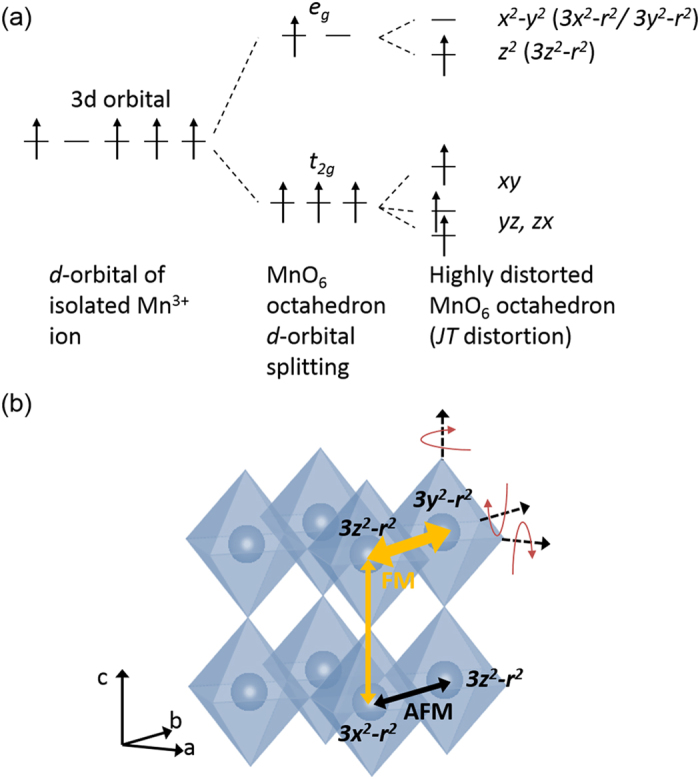
(**a**) Splitting in energy of the *d*-orbitals of Mn^3+^ due to the MnO_6_ octahedron and the further Jahn-Teller distortion (**b**) Schematic illustration of the magnetic interactions of Mn^3+^ ions (blue balls) with *e*_*g*_ orbitals within MnO_6_ octahedra in BiMnO_3_. The Bi^3+^ ions at the *A*-site are not shown. Oxygen is at the corners of the octahedra. Yellow arrows and black arrow indicate ferromagnetic and antiferromagnetic superexchange interactions, respectively. The thickness of arrows indicates the strength of the couplings^9^. Red arrows show MnO_6_ octahedral rotations.

**Figure 2:**
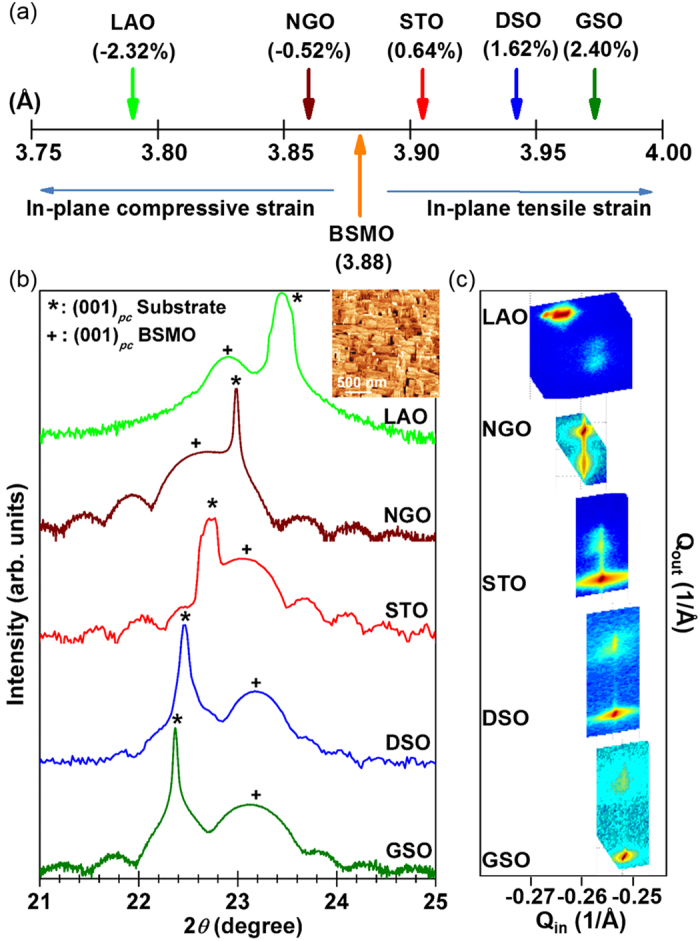
(**a**) Lattice mismatch between BSMO and the various substrates at RT. XRD spectra of ~20 nm thick BSMO films grown on the various substrates: (**b**) The BSMO (001) peak and substrate peak are marked by (+) and (*), respectively. Reflections are indexed based on pseudo-cubic symmetry. The inset shows an atomic force microscopy image with 2 × 2 μm^2^ for BSMO grown on STO. The root-mean-square (RMS) roughness of the film was ∼0.26 nm. (**c**) RSMs around the (332) peak of DSO, GSO and NGO, and (103) of STO and LAO. The substrates peaks are the sharper, red-centred, high intensity peaks which appear at the bottom of the RSM plots, except for LAO and NGO.

Taking the above information into account, for strong tensile strained orthorhombic-like BSMO (*c/a* < 0.98), stronger *in-plane* FM interactions are exepcted (see Fig. 1b) since the *in-plane* bond angles are likely to be relatively close to 180°, as a result of the weak rotations along the *out-of-plane* axis.

Looking at the left hand region of Fig. 4, this is indeed observed, i.e. the high *T*_*C*_ samples show clear FM transitions. The results agree with literature, where rare earth manganites and cobaltites show stronger FM interactions and higher *T*_*C*_s when the bond angles are close to 180° 36,37.

**Figure 3:**
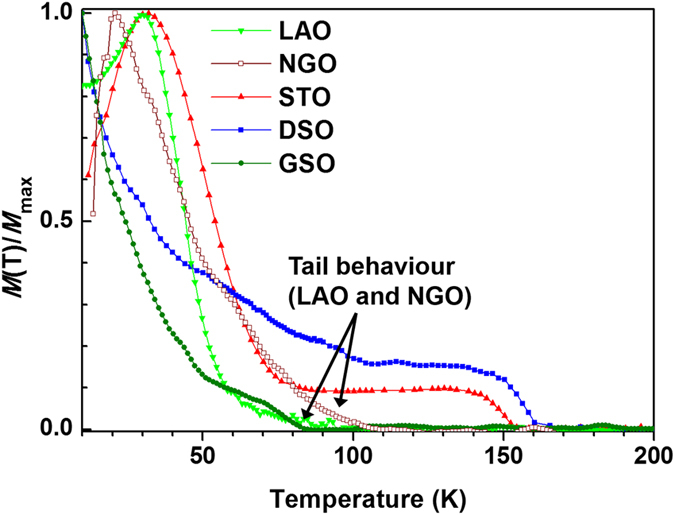
The temperature dependence of magnetisation (*M *− *T*) for ~20 nm BSMO films grown on various substrates. The normalized *in-plane M* − *T* curves of BSMO on STO and LAO were measured at a field of 200 Oe. The *M* − *T* curves of BSMO on DSO, GSO and NGO were measured at 0 Oe after field cooling at 1 T. Arrows indicate the tail behaviour present for LAO and NGO substrates.

The highest *T*_*C*_ (~160 K) was found for BSMO on DSO (*c/a* = 0.967 ± 0.005). A reduced *T*_*C*_ of ~83 K was found for BSMO on GSO. This film would be expected to have the highest *T*_*C*_ based on it having the lowest *c/a* ratio. However, it is very likely that this film has an increased defect concentration because of the very large *in-plane* strain which results in a significant increase in the unit-cell volume (Table 1). For the films on DSO and GSO, the *in-plane* FM interaction becomes stronger and dominant, and so the AFM signal becomes relatively weak because the AFM interaction is hidden by the strong FM signal. This is the same situation as in bulk BMO where only FM behaviour is observed. Instead of showing a clear AFM behaviour, i.e. an AFM transition and/or a tail behaviour, the *M* − *T* curves for films on DSO and GSO are not saturated at low temperature (see Fig. 3). This lack of saturation is indicated by remanent AFM components, which could originate from a cluster–glass magnetic state^28^.

Even though there is remanent AFM behaviour, the *M*_*r*_ of BSMO on DSO has the largest value (~0.18 μ_B_/Mn). This is because of a stronger FM behaviour arising from dominant FM interactions. On the other hand, BSMO on GSO has the lowest *M*_*r*_ (~0.06 μ_B_/Mn), consistent with increased defects in the film.

**Figure 4:**
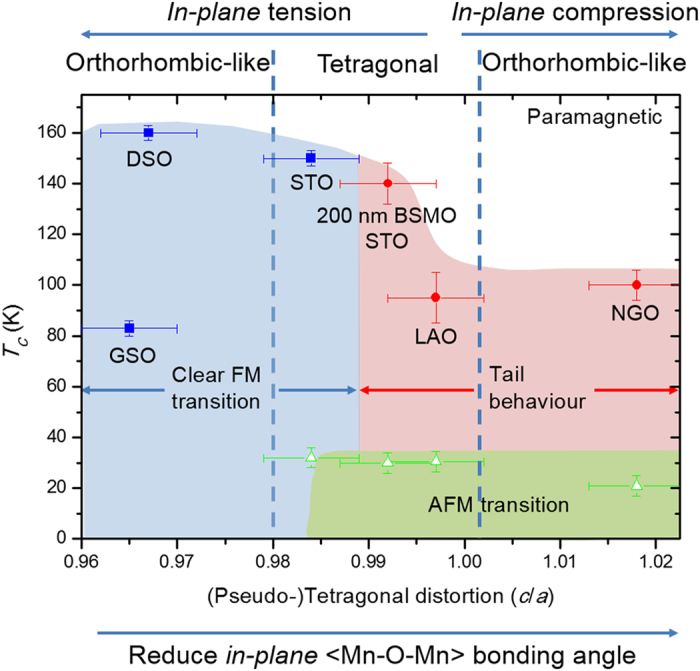
Magnetic phase diagram for BSMO showing the relationship between tetragonality of pseudo-cubic and the magnetic transition temperature based on ~20 nm thick BSMO films studied in this paper. The data for 200 nm thick BSMO on STO (from ref. 18) is included to provide more information about the transition region near *c/a* ~ 0.99.

Looking at the middle region (0.98 < *c/a* < 1) of Fig. 4 where there is a low level of *in-plane* tension, the BSMO is tetragonal instead of orthorhombic-like as was observed for high *in-plane* tension (0.98 < *c/a*). Since *in-plane* rotations are absent for this low strain level, there are stronger *out-of-plane* FM interactions, with an *out-of-plane* 〈Mn-O-Mn〉 bond angle of 180°. This leads to an intermediate magnetic state and explains why for STO and LAO AFM interactions (at ~30 K) are observed in addition to FM interactions (see Fig. 3).

Finally, looking at the right hand region (*c/a* > ~1) of Fig. 4 where there is a higher lever of *in-plane* compressive strain, an orthorhombic-like structure is produced once more for BSMO on NGO. Hence, in this region, stronger *out-of-plane* FM interactions and weaker *in-plane* FM interactions are expected. Therefore, the *in-plane* FM interactions do not dominate the magnetic behaviour. As a result, *T*_*C*_ decreases as the *c/a* ratio is increased, and a clear AFM transition is observed around 30 K, as well as an AFM tail behaviour (see Fig. 3).

**Figure 5:**
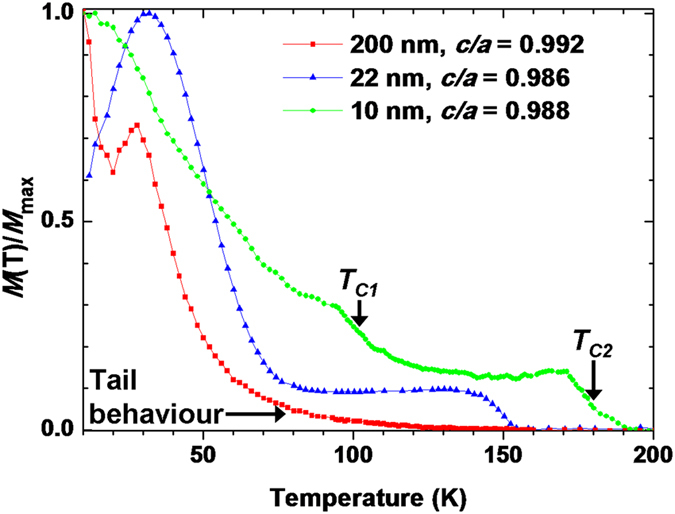
The normalized *in-plane M* − T **curves for BSMO thin films on STO with different thickness, 200 (taken from ref.** 1820 and 10** nm at a field of 200 Oe.**

Finally, by studying different thickness films on STO, the intermediate transition region (0.980 < *c/a* < 0.995) of Fig. 4 is probed more closely. Figure 5 shows the *M* − *T* curves for BSMO films on STO of 200, 20 and 10 nm thickness. The different film thicknesses result in different levels of strain relaxation and hence different *c/a* ratios. For the 200 nm thick film, the *T*_*C*_ is enhanced to ~140 ± 6 K (*cf*. the bulk BMO *T*_*C*_ of ~100 K). Both a tail behaviour and an AFM transition at 30 K are observed but not a clear FM transition. For the 20 nm thick film, a clear FM transition is observed with *T*_*C*_ of 150 ± 3 K. There is no tail behaviour, but there is an AFM transition at ~30 K.

For the 10 nm thick film, a clear FM transition is observed with *T*_*C*_ (termed *T*_*C2*_) of 176 ± 3 K. There is no tail behaviour, nor a 30 K AFM transition. However, there is a second FM transition with *T*_*C*_ (termed *T*_*C1*_) at 100 ± 3 K. *T*_*C2*_ is surprisingly large, enhanced by ~70 K compared to bulk BMO, while *T*_*C1*_ is very close to the *T*_*C*_ of bulk BMO. This feature is similar to ultrathin La_0.67_Sr_0.33_MnO_3_ (LSMO) films (<10 unit cells) where a double transition is found with a much enhanced *T*_*C*_ at ~560 K, and a second lower transition at ~200 K. Thicker LSMO films (>20 unit cells), on the other hand, show bulk *T*_*C*_s of ~350 K^27^. The two ferromagnetic phases for the 10 nm thick BSMO film likely originate from one or more of the possible strain relaxation mechanisms which occur in perovskite films, e.g. phase separation, non-stoichiometry, or deformation of *B*O_6_ octahedra^38^,^39^,^40^,^41^,^42^,^43^.

In conclusion, the relation of magnetic properties and crystal structure in BiMnO_3_, the most promising multiferroic with a relatively high FM *T*_*C*_, was determined for the first time. Sm-doping of the Bi site at a level of 15% was undertaken to reduce the unit cell volume to enhance FM coupling (3D chemical pressure), and different substrates were used (2D biaxial pressure) to expand or contract the unit cell *in-plane*. Hence, 3D + 2D biaxial straining were used *together* to precisely engineer the BiMnO_3_ crystal structure, so as to optimise the 3D Mn-O-Mn magnetic coupling. A magnetic phase diagram for the system was mapped out for 0.96 < c < 1.02. It was possible to eliminate the hidden AFM and strongly increase the *T*_*C*_ by optimizing the (pseudo-)tetragonal distortion (*c/a*). For *c/a* < 0.98, the films showed a clear FM behaviour. On the other hand, for *c/a* > 0.98, hidden AFM was revealed in addition to the FM behaviour.

With appropriate choice of substrate, doping and film thickness, it was possible to strongly increase *T*_*C*_. A 10 nm thick Sm-doped BiMnO_3_ film grown on STO showed a sharp FM *T*_*C*_ of 176 ± 3 K and a 20 nm thick Sm-doped BiMnO_3_ film grown on DSO showed a sharp FM *T*_*C*_ of 160 ± 3 K. Of course, the ultimate goal for BMO films is to achieve both strong FM and FE, and to do this above room temperature. To achieve this, the right strain state needs to be engineered into films thicker than a few 10’s of nm, thereby reducing leakage. A possible future approach is to use nanocomposite films where high strain levels can be induced in thick films (>100 nm) and where enhanced FM and FE properties have already been demonstrated in other perovskites^44^.

## Methods

Films were grown by pulsed laser deposition (PLD) from a single ceramic target. The growth rate was 0.07-0.08 Å/pulse. For the film growth, the laser pulse rate was 2 Hz with a laser fluence of 1.3 Jcm^−2^. The oxygen pressure was fixed at 100 mTorr and growth temperature was 650 °C. To confirm the phase purity, crystalline quality and the 3D strain state of the films at RT, 2*θ* − *ω* scans and reciprocal space maps (RSMs) were carried out using a Panalytical Empyrean high resolution x-ray diffraction (XRD) system. We determined the *out-of-plane* lattice parameter by measuring the position of the (00 *l*) peaks in the *2θ* − *ω* scans as well as the (103) pseudo-cubic (pc) peak of the BSMO in the RSMs. The film thicknesses were determined by x-ray reflectivity. To determine the film surface morphology, atomic force microscopy was performed. The temperature dependence of magnetisation (*M* − *T*) was determined using a superconducting quantum interference device (SQUID) magnetometer (Quantum Design, MPMS).

## Additional Information

**How to cite this article:** Choi, E.-M. *et al*. Strain-tuned enhancement of ferromagnetic *T_C_* to 176 K in Sm-doped BiMnO_3_ thin films and determination of magnetic phase diagram. *Sci. Rep.* **7**, 43799; doi: 10.1038/srep43799 (2017).

**Publisher's note:** Springer Nature remains neutral with regard to jurisdictional claims in published maps and institutional affiliations.

## Supplementary Material

Supplementary Information

## Figures and Tables

**Table 1:** Pseudo-cubic unit cell lattice parameters, lattice mismatch, unit cell volume, (pseudo−) tetragonal distortion, crystal structure, magnetic transition temperature (*T*_*C*_) and remanent magnetic moment (*M*_*r*_) at 10 K of ~20 nm BSMO thin films grown on various substrates.

Substrate	*In-plane* lattice parameter Substrate [Å]	Lattice mismatch (%)	*In-plane* lattice parameter BSMO (Å)	*Out-of-plane* lattice parameter BSMO [Å]	Pseudo−cubic unit cell volume [Å^3^]	(Pseudo−) Tetragonal distortion, *c/a*	Crystal Structure BSMO	*T*_*C*_ (K)	*Mr* (μ_B_/Mn) at 10 K
LAO(001)	3.790	−2.32	3.89 ± 0.005	3.88 ± 0.005	58.7 ± 0.2	0.997 ± 0.005	Tetragonal	95 ± 10	0.10
NGO(110)^a^	3.864	−0.52	3.86 ± 0.005	3.93 ± 0.005	58.6 ± 0.2	1.018 ± 0.005	Orthorhombic	100 ± 6	0.11
STO (001)	3.905	0.64	3.90 ± 0.005	3.845 ± 0.005	58.6 ± 0.2	0.984 ± 0.005	Tetragonal	150 ± 3	0.12
DSO(110)^a^	3.943	1.62	3.94 ± 0.005	3.81 ± 0.005	59.1 ± 0.2	0.967 ± 0.005	Orthorhombic	160 ± 3	0.18
GSO(110)^a^	3.973	2.40	3.97 ± 0.005	3.83 ± 0.005	60.4 ± 0.2	0.965 ± 0.005	Orthorhombic	83 ± 3	0.06

^a^Average pseudo-cubic *in-plane* lattice parameter of orthorhombic structure determined from the RSMs.
