# Supplementary material for: Strain-tuned enhancement of ferromagnetic TC to 176 K in Sm-doped BiMnO3 thin films and determination of magnetic phase diagram
Source: Sci Rep. 2017 Mar 3;7:43799. doi: 10.1038/srep43799 (PMC5335565; doi:10.1038/srep43799)
Supplement: Supplementary Information [file srep43799-s1.pdf]

Supplementary Information

Strain-tuned enhancement of ferromagnetic T_C to 176 K in Sm-doped $BiMnO_3$ thin films and determination of magnetic phase diagram

Eun-Mi Choi*, Josée E. Kleibeuker and Judith L. MacManus-Driscoll

Asymmetric Reciprocal space maps of (103) pseudo-cubic (pc) peak of the BSMO at various phi-angles.

Tetragonal structure.

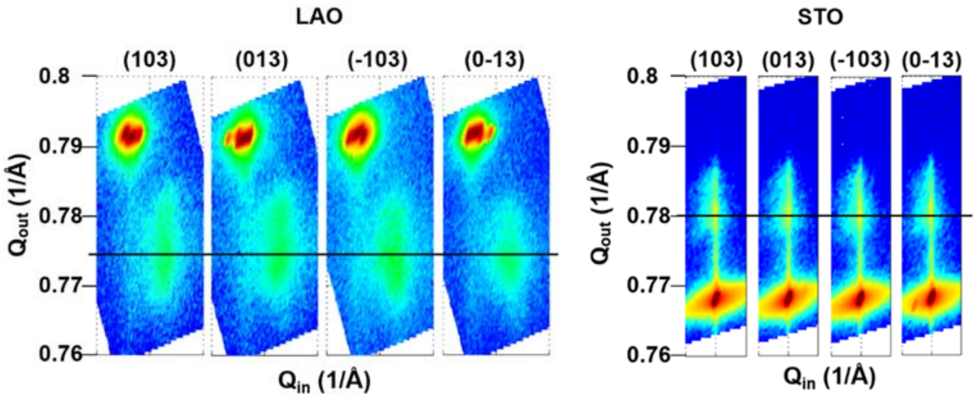

Orthorhombic-like Structure.

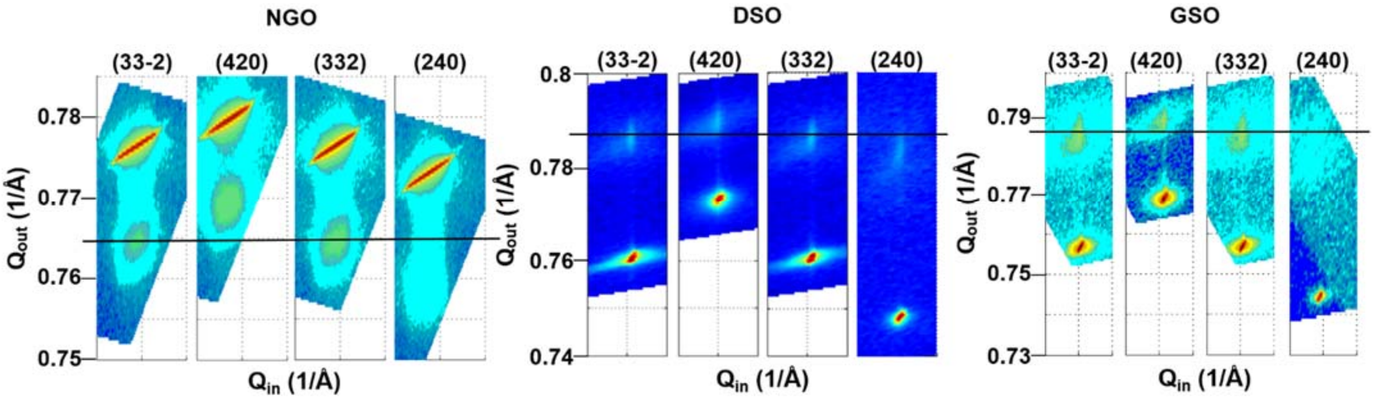

Solid lines are guide for eyes.
